# Cyberbullying: Study of a series of 96 Moroccan adolescents and young adults

**DOI:** 10.1192/j.eurpsy.2023.1825

**Published:** 2023-07-19

**Authors:** Y. El Hilali, F. Manoudi

**Affiliations:** 1 Child psychiatry; 2 Psychiatry, University Hospital Mohammed VI of Marrakech, Marrakech, Morocco

## Abstract

**Introduction:**

Cyberbullying is a form of virtual harassment through the Internet, cell phones and social networks. Its psychopathological consequences are serious, including the risk of suicide.

**Objectives:**

The aim of this study is to determine the prevalence of cyberbullying in a sample of Moroccan adolescents, as well as the associated comorbidities.

**Methods:**

This is a descriptive cross-sectional study of cyberbullying among Moroccan adolescents and young adults aged 12 to 20 years old. The data collection was done by a survey on Google Form sent on social networks

**Results:**

A number of 120 forms were completed by high school students, followed by secondary school and university students, of which 96 were valid. The average age was 16 years with a sex ratio of 0.25. Fifty-nine percent of the youth spent more than two hours daily on the Internet and 73% admitted having been bullied online at least once while 19% said it happened often. The profile of harassers included strangers, followed by acquaintances and schoolmates. The platforms where harassment was most present were Facebook, Instagram and WhatsApp. The most frequent types of cyberbullying were private hate messages, humiliating comments about physical appearance and messages with sexual connotations. After the harassment, 65% of victims did not tell anyone; 68% of victims felt angry, and one in five felt suicidal. Among the most common comorbidities were adjustment disorder, anxiety and depressive symptoms, aggressive behavior, and suicidal ideation. Half of the youth felt that cyberbullying had impacted their school or family life.

**Conclusions:**

Parents, educators, and health professionals need to be aware of the risks of virtual communication, and the link between cyberbullying and mental health disorders, and to develop national intervention and prevention programs.

**Disclosure of Interest:**

None Declared

